# Pretreatment Cerebrospinal Fluid Bacterial Load Correlates With Inflammatory Response and Predicts Neurological Events During Tuberculous Meningitis Treatment

**DOI:** 10.1093/infdis/jiy588

**Published:** 2018-10-09

**Authors:** Nguyen T T Thuong, Dao N Vinh, Hoang T Hai, Do D A Thu, Le T H Nhat, Dorothee Heemskerk, Nguyen D Bang, Maxine Caws, Nguyen T H Mai, Guy E Thwaites

**Affiliations:** 1 Oxford University Clinical Research Unit, Ho Chi Minh City, Viet Nam; 2 Pham Ngoc Thach Hospital, Ho Chi Minh City, Viet Nam; 3 Hospital for Tropical Diseases, Ho Chi Minh City, Viet Nam; 4 Nuffield Department of Medicine, University of Oxford, United Kingdom; 5 Liverpool School of Tropical Medicine, United Kingdom

**Keywords:** Bacterial load, tuberculous meningitis, inflammatory response, cytokines, neurological events

## Abstract

**Background:**

The *Mycobacterium tuberculosis* load in the brain of individuals with tuberculous meningitis (TBM) may reflect the host’s ability to control the pathogen, determine disease severity, and determine treatment outcomes.

**Methods:**

We used the GeneXpert assay to measure the pretreatment *M. tuberculosis* load in cerebrospinal fluid (CSF) specimens from 692 adults with TBM. We sought to understand the relationship between CSF bacterial load and inflammation, and their respective impact on disease severity and treatment outcomes.

**Results:**

A 10-fold higher *M. tuberculosis* load was associated with increased disease severity (odds ratio, 1.59; *P* = .001 for the comparison between grade 1 and grade 3 severity), CSF neutrophil count (r = 0.364 and *P* < .0001), and cytokine concentrations (r = 0.438 and *P* < .0001). A high *M. tuberculosis* load predicted new neurological events after starting treatment (*P* = .005, by multinomial logistic regression) but not death. Patients who died had an attenuated inflammatory response at the start of treatment, with reduced cytokine concentrations as compared to survivors. In contrast, patients with high pretreatment CSF bacterial loads, cytokine concentrations, and neutrophil counts were more likely to subsequently experience neurological events.

**Conclusions:**

The pretreatment GeneXpert-determined *M. tuberculosis* load may be a useful predictor of neurological complications occurring during TBM treatment. Given the evidence for the divergent pathogenesis of TBM-associated neurological complications and deaths, therapeutic strategies to reduce them may need reassessment.

## Teaser

In tuberculous meningitis (TBM), a higher pretreatment CSF bacterial load was associated with increased disease severity and host inflammation. The bacterial load can be used to predict new neurological events but not death. Owing to the divergent pathogenesis of TBM-associated neurological complications and deaths, strategies to reduce them may need reassessment.

Tuberculous meningitis (TBM) is the most severe form of tuberculosis. It is caused by dissemination of *Mycobacterium tuberculosis* to the brain, resulting in meningoencephalitis with necrotizing, granulomatous inflammation predominantly affecting the basal meninges. Inflammation can lead to life-threatening complications of hydrocephalus, infarcts, and tuberculomas [1, 2]. Death or neurological disability still occur in half of all cases.

The pathogenesis of TBM is not well understood. Much of the pathology is thought to arise from the immune response to replicating bacteria, but the nature of that response and its relationship to bacterial numbers is presently poorly defined [3]. Recent studies in large, well-characterized cohorts of human immunodeficiency virus (HIV)–uninfected Vietnamese adults with TBM demonstrated that elevated cerebrospinal fluid (CSF) concentrations of 8 of 10 tested cytokines were associated with more-severe disease [4]. Death, however, was associated with an attenuated inflammatory response, lower CSF cytokine concentrations and leukocyte counts [4], and higher CSF neutrophil counts [5]. HIV infection and polymorphism rs17525495 in the gene encoding leukotriene A4 hydrolase (*LTA4H*) influenced both pretreatment CSF inflammatory phenotype and survival from TBM in Vietnamese individuals [4], but the polymorphism was not associated with survival in Indonesians [5].

Neutrophils appear to play an important role in TBM pathogenesis [5]. Higher CSF neutrophil numbers have been associated with an increased likelihood of culturing *M. tuberculosis* from the CSF [6, 7] and of cerebral immune reconstitution inflammatory syndrome (IRIS) in those coinfected with HIV [8, 9].

One of the fundamental questions concerning TBM pathophysiology is how the *M. tuberculosis* load relates to intracerebral inflammation and outcome. Very low bacterial numbers in CSF and inadequate laboratory methods have to date made this question largely refractory to investigation. CSF culture positivity may indicate higher bacterial loads and was associated with mortality in HIV-uninfected patients with TBM [5]. More-recent molecular assays, such as GeneXpert, quantify bacterial nucleic acid in CSF and are now widely used for TBM diagnosis. Although GeneXpert’s sensitivity for TBM diagnosis is around 60% as compared to clinical diagnosis [10], the bacterial load in CSF specimens that test positive can be categorized as very low, low, medium, or high, thus offering a new way to assess the CSF *M. tuberculosis* load in clinical practice. The aim of our study was to use GeneXpert to define the CSF bacterial load in a large cohort of well-characterized Vietnamese adults with TBM and to investigate the relationship between bacterial load and CSF cytokine concentrations, leukocyte numbers and types, and the occurrence of new neurological events and death after the start of antituberculosis treatment.

## METHODS

### Participants

Adults (age, >17 years) with TBM were enrolled into a randomized controlled trial of intensified antituberculosis chemotherapy between April 2011 and June 2014 [11]. Of the 817 trial participants, 692 had CSF GeneXpert data and were included in the current study. The other 125 participants had missing data because of an insufficient volume of CSF samples or because of GeneXpert test errors.

Written informed consent was obtained from each participant or from an accompanying relative if the participant could not provide consent. Protocols were approved by the Oxford Tropical Research Ethics Committee in the United Kingdom, the institutional review boards of the Hospital for Tropical Diseases and Pham Ngoc Thach Hospital for Tuberculosis and Lung Disease, and the Ethical Committee of the Ministry of Health in Vietnam.

### Treatment

Participants were randomly allocated to treatment with (1) a standard antituberculosis regimen for 3 months, followed by rifampicin and isoniazid at the same doses for a further 6 months, or (2) an intensified regimen that consisted of the standard regimen with an additional higher dose of rifampicin (15 mg/kg/day) and levofloxacin (20 mg/kg/day) for the first 8 weeks of treatment. All participants also received adjunctive dexamethasone for the first 6–8 weeks of treatment [11].

### Clinical and CSF Characteristics

We extracted data on participant age, weight, duration of illness (days of symptoms), Glasgow coma scale (GCS), British Medical Research Council (BMRC) grading for TBM, antituberculosis treatment, and HIV status on the date of TBM diagnosis. All participants were followed for 9 months, with careful characterization of their clinical response to treatment. New neurological events were defined as the new occurrence of any of the following: cerebellar symptoms; mono-, hemi-, para, or tetraplegia; seizures; cranial nerve palsy; or a ≥2-point decrease in the GCS for ≥2 days after the highest previously recorded score [11]. These events encompass paradoxical treatment reactions, which are thought to be caused by an excessive inflammatory response to dead or dying bacteria [12] and by death of brain tissue.

Laboratory data were collected from samples collected on the date of enrollment and included blood and CSF cell counts and cytokine concentrations. A panel of 10 cytokines important in inflammation in tuberculosis, comprising 6 proinflammatory cytokines (interleukin 1β [IL-1β], interleukin 2 [IL-2], interleukin 6 [IL-6], interleukin 12p70 [IL-12p70], interferon γ [IFN-γ], and tumor necrosis factor α [TNF-α]) and 4 antiinflammatory cytokines (interleukin 4 [IL-4], interleukin 5 [IL-5], interleukin 6 [IL-6], and interleukin 13 [IL-13]), was measured in stored CSF specimens, using the Luminex multiplex bead-based assay [4]. The *LTA4H* rs17525495 polymorphism was genotyped by the TaqMan genotyping assay [3].

GeneXpert MTB/RIF, which detects *M. tuberculosis* complex and rifampicin resistance, was performed on CSF specimens before the start of antituberculosis treatment [10]. Briefly, 3–5 mL of CSF was centrifuged for 20 minutes at 3000*×g;* 200 μL (about one third of the deposit) was resuspended in phosphate-buffered saline and mixed with 1.5 mL of sample reagent, vortexed for 30 seconds, incubated for 15 minutes at room temperature, and then transferred to a GeneXpert cartridge for measurement. The GeneXpert Dx software (version 4.0; Cepheid) reported semiquantitative mycobacterial load results as cycle threshold (Ct) values, representing the number of PCR cycles required for the signal to reach a detection threshold. *M. tuberculosis* loads were classified as high (Ct value, <16), medium (16–22), low (22–28), and very low (>28). *Mycobacterium bovis* (bacillus Calmette-Guérin; NCTC 5692) was used to generate a standard curve of GeneXpert Ct values versus bacterial numbers by the Miles and Misra method [13]. For each sample, the average Ct value of 5 probes (excluding any delayed values due to rifampicin resistance) was used to estimate the bacterial load and analyze further.

### Statistical Analysis

Data were analyzed using R, version 3.0.1 [14], MATLAB and Statistics Toolbox, release 2013a (MathWorks, Natick, MA), and GraphPad Prism, version 6 (GraphPad Software, San Diego, CA). In statistical analyses, to preserve statistical power we used continuous comparisons of the whole data set, with negative results of GeneXpert assigned a Ct value of 40; most of the graphical representations present bacterial loads in categories, for clarity.

Baseline concentrations of the 10 CSF cytokines were analyzed using principal component analysis (PCA), a method of transforming complex correlated data sets into a new coordinator system (ie, the component space) in which 2 or 3 first principal components (PCs) can cover up to 70%–80% variance of the whole data set. By this means we visualized our multivariate data set and avoided multiple comparisons [15, 16]. Associations between *M. tuberculosis* load and cytokine PCs, blood counts, or CSF cell counts were examined using the Spearman rank correlation coefficient, in which log-transformed cell counts were used to analyze correlations. Comparisons of bacterial loads by GCS or disease severity, CSF neutrophil counts, and cytokines were performed by a linear trend test implemented using robust linear regression. We classified outcomes into 3 groups: (1) “survival,” defined as survival without new neurological events; (2) “new neurological events,” defined as survival with new neurological events occurring during treatment; and (3) “death,” defined as death during treatment. Comparisons of the bacterial load between the survival group and the new neurological events or death groups used Mann-Whitney tests. The outcome was modeled using multinomial logistic regression depending on *M. tuberculosis* bacterial load and the detection limit indicator. The model was later adjusted for predefined risk factors, including age, weight, GCS, CSF leukocyte numbers, antituberculosis regimen, antiretroviral therapy, HIV infection, and *LTA4H* genotype. Separate analysis was also performed for each HIV status. We used the Hochberg method to correct *P* values for multiple testing.

## RESULTS

### Characteristics of Participants

The baseline clinical characteristics of the 692 adults with TBM for whom the CSF *M. tuberculosis* load was assessed by GeneXpert are described in Table 1. A total of 293 (42.3%) were GeneXpert positive, and these individuals were significantly more likely than the GeneXpert-negative participants to be HIV infected and to have more-severe disease. The majority of participants (568 [82.1%]) had BMRC grade 1 or 2 disease at enrollment. *M. tuberculosis* was detected in CSF specimens from 257 participants (37.6%) and 281 participants (41.2%) by microscopy and mycobacterial growth indicator tube (MGIT) culture, respectively. In the 228 patients with positive results of both GeneXpert and MGIT culture, correlation between the bacillary load and the days to culture positivity was 0.417 (*P* < .0001). Participants who were GeneXpert positive had greater numbers of leukocytes, percentages of neutrophils, and levels of protein and lactate in CSF than those who were GeneXpert negative. Clinical characteristics of HIV-uninfected patients and HIV-infected patients and their blood and CSF parameters are shown in Supplementary Table 1.

**Table 1.** Baseline Clinical Characteristics of All 692 Participants With Tuberculous Meningitis (TBM), Overall and by Cerebrospinal Fluid GeneXpert Result

Characteristic	Overall		GeneXpert Negative		GeneXpert Positive		*P* ^a^
	No.^b^	Summary Statistic	No.^b^	Summary Statistic	No.^b^	Summary Statistic	
Age, y	692	35 (29–46)	399	36 (29–50)	293	35 (29–42)	.008
Male sex	692	475 (68.6)	399	261 (65.4)	293	214 (73.0)	.038
Weight, kg	692	48 (44–54.5)	399	49 (45–55)	293	48 (43–54)	.144
Duration of illness, d	692	15 (10–30)	399	15 (10–30)	293	15 (10–30)	.763
Glasgow coma scale	692	15 (12–15)	399	15 (13–15)	293	14 (11–15)	<.0001
HIV infected, no. (%)	692	288 (41.6)	399	123 (30.8)	293	165 (56.3)	<.0001
Standard treatment arm	692	345 (49.9)	399	199 (49.9)	293	146 (49.8)	1
BMRC grade^c^	692	…	399	…	293	…	.001
I	…	266 (38.4)	…	162 (40.6)	…	104 (35.5)	…
II	…	302 (43.6)	…	184 (46.1)	…	118 (40.3)	…
III	…	124 (17.9)	…	53 (13.3)	…	71 (24.2)	…
Other diagnostic tests							
CSF smear positive	684	257 (37.6)	394	69 (17.5)	290	188 (64.8)	<.0001
CSF MGIT culture positive	682	281 (41.2)	389	53 (13.6)	293	228 (77.8)	<.0001
Diagnostic category^d^	692		399		293		<.0001
Definite TBM	…	397 (57.4)	…	104 (26.1)	…	293 (100)	…
Probable TBM	…	170 (24.6)	…	170 (42.6)	…	0 (0)	…
Possible TBM	…	125 (18.0)	…	125 (31.3)	…	0 (0)	…
CSF parameter							
Total leukocyte count, ×10^3^ cells/mL	686	115 (35–284)	395	76 (22–198)	291	200 (64–388)	<.0001
Neutrophils, %	655	10 (0–32)	371	3 (0–15)	284	20 (5–56)	<.0001
Lymphocytes, %	656	90.0 (68–100)	372	97.5 (85–100)	284	79.5 (44–95)	<.0001
Protein level, g/L	666	1.2 (0.6–1.9)	379	1.0 (0.5–1.7)	287	1.4 (0.9–2.4)	<.0001
Glucose level, mmol/L	666	1.9 (1.3–2.7)	379	2.2 (1.4–3.0)	287	1.6 (1.0–2.2)	<.0001
Lactate level, mmol/L	638	4.8 (3.5–6.5)	357	4.1 (2.9–5.6)	281	5.9 (4.5–7.4)	<.0001

Data are no. (%) of participants or median value (interquartile range).

Abbreviations: CSF, cerebrospinal fluid; HIV, human immunodeficiency virus; MGIT, mycobacterial growth indicator tube.

^a^ *P* values are descriptive only and based on the χ^2^ test (for categorical data) and the Mann-Whitney test (for continuous data).

^b^Data denote the number of patients with nonmissing data for the corresponding variable.

^c^Modified British Medical Research Council (BMRC) grade I indicates a GCS of 15 with no neurologic signs (baseline), grade II indicates a score of 11–14 (or 15 with focal neurologic signs), and grade III indicates a score of ≤10.

^d^Diagnostic categories were assigned according to the consensus case definition [17]. Patients with an unlikely diagnosis of TBM had a score of <6. Confirmed other diagnosis was only made on the basis of microbiological evidence.

**Figure 1.**
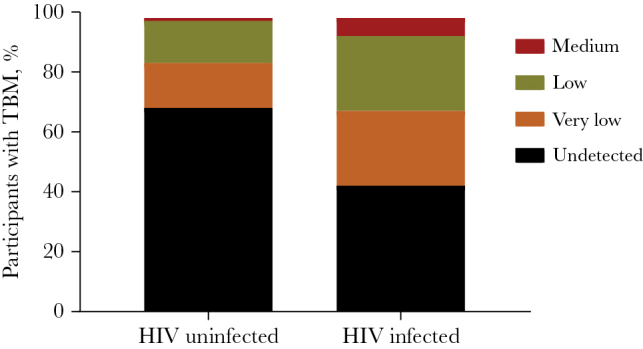
Bacterial load in 692 participants with tuberculous meningitis who were (n = 288) or were not (n = 404) infected with human immunodeficiency virus (HIV) before the start of antituberculosis treatment. Data are percentages of participants with undetectable, very low, low, and medium *Mycobacterium tuberculosis* levels.

The frequency of *M. tuberculosis* detection by GeneXpert in HIV-infected participants (165 of 288 [57.3%]) was higher than in HIV-uninfected participants (128 of 404 [31.7%]; *P* < .0001), although *M. tuberculosis* loads in these 2 groups were not significantly different (*P* = .087). Pretreatment GeneXpert CSF bacterial loads varied from very low to medium; a high load was not recorded. In the 404 HIV-uninfected participants, 276 (68.3%) had a negative test result, 63 (15.6%) had very low bacterial loads, 58 (14.4%) had very low bacterial loads, and 7 (1.7%) medium bacterial loads. In the 288 HIV-infected participants, 123 (42.7%) had a negative test result, 72 (25.0%) had very low bacterial loads, 74 (25.7%) had low bacterial loads, and 19 (6.6%) had medium bacterial loads (Figure 1). Conversions from Ct values to bacterial colony-forming units (CFUs) and ranges of bacterial counts corresponding to bacterial load categories can be found in Supplementary Figure 1.

### Association Between Pretreatment *M. tuberculosis* Load and Disease Severity

**Figure 2.**
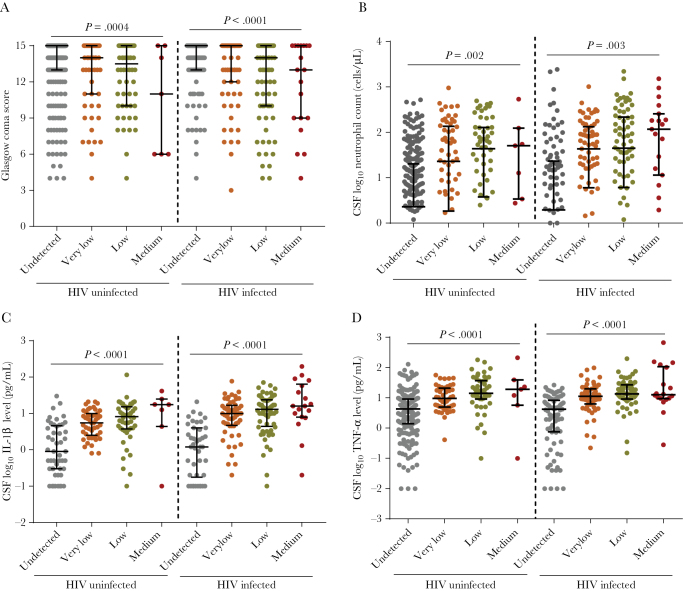
Associations between *Mycobacterium tuberculosis* levels at baseline and Glasgow coma scale (*A*), cerebrospinal fluid (CSF) neutrophil count (*B*), CSF interleukin 1β (IL-1β) concentration (*C*), and CSF tumor necrosis factor α (TNF-α) concentration (*D*) before the start of antituberculosis treatment among participants with tuberculous meningitis. Data are for 404 human immunodeficiency virus (HIV)–uninfected patients (276 with undetectable, 63 with very low, 58 with low, and 7 with medium bacterial levels) and 288 HIV-infected participants (123 with undetectable, 72 with very low, 74 with low, and 19 with medium bacterial levels). *P* values were derived from the linear trend test. Long bars in plots represent median values, and short bars represent interquartile ranges.

The associations between *M. tuberculosis* load and GCS or BMRC grade were examined before treatment in 692 adults with TBM. *M. tuberculosis* loads showed positive correlations with GCS (r = 0.194 and *P* < .0001 for the HIV-uninfected group, and r = 0.238 and *P* < .0001 for the HIV-infected group, both by Spearman correlation). Specifically, higher bacterial loads were observed in individuals with more severe coma, and an increased *M. tuberculosis* load was associated with a lower GCS (*P* = .0004 for the HIV-uninfected group, and *P* < .0001 for the HIV-infected group, by the test for linear trend; Figure 2A).

In addition, an increased *M. tuberculosis* load was also associated with more-severe disease, as assessed by BMRC grade (*P* = .008 for the HIV-uninfected group and *P* = .001 for the HIV-infected group, both by the test for linear trend). The bacterial load (per 10-fold increase) affected disease severity in all participants. For all participants, the odds ratio (OR) was 0.71 (95% confidence interval [CI], .91–1.50; *P* = .226) for grade 1 versus grade 2 and 1.59 (95% CI, 1.19–2.15; *P* = .001) for grade 1 versus grade 3. For HIV-uninfected participants, the OR was 1.56 (95% CI 1.02–2.47; *P* = .039) for grade 1 versus grade 2 and 1.84 (95% CI, 1.12–3.21; *P* = .015) for grade 1 versus grade 3. For HIV-infected participants, the OR was 1.0 (95% CI, .7–1.4; *P* = .878) for grade 1 versus grade 2 and 1.5 (95% CI, 1.1–2.2; *P* = .013) for grade 1 versus grade 3. The duration of illness before treatment was not associated with BMRC grade (*P* = .977) or *M. tuberculosis* load (*P* = .651).

### Correlations Between Pretreatment CSF *M. tuberculosis* Load and Inflammation

To investigate whether the *M. tuberculosis* load before treatment was related to inflammatory responses, we first explored the relationship between *M. tuberculosis* load and blood and CSF cell counts. Bacterial loads stratified by Ct values showed negative correlations with neutrophil numbers in blood and CSF specimens from both HIV-infected participants and uninfected participants (Table 2 and Figure 2B). These correlations were stronger in CSF (r = −0.395 and *P* < .0001; Supplementary Figure 3) than in blood (Table 2), suggesting that neutrophil recruitment correlated more strongly with bacterial replication at the infected site.

**Table 2.** Relationship of *Mycobacterial tuberculosis* Load and White Blood Cell Numbers and Cytokine Levels in Blood and Cerebrospinal Fluid Specimens From Patients With Tuberculous Meningitis, by Spearman Correlation

Group	Blood			Cerebrospinal Fluid						
	Leukocytes	Lymphocytes	Neutrophils	Leukocytes	Lymphocytes	Neutrophils	IL-1β	TNF-α	PC1	PC2
**Overall, no.**	650	649	650	682	653	651	521	522	518	518
r^a^	-0.027	**0.242** ^**b**^	-0.085	**-0.273** ^**b**^	**-0.11** ^**b**^	**-0.395** ^**b**^	**-0.523** ^**b**^	**-0.48** ^**b**^	**-0.493** ^**b**^	**0.185** ^**b**^
*P*	.486	< .0001	.031	< .0001	.005	< .0001	< .0001	< .0001	< .0001	< .0001
Adjusted *P*^c^	.486	< .0001	.062	< .0001	.014	< .0001	< .0001	< .0001	< .0001	< .0001
**HIV uninfected, no.**	382	382	382	401	390	389	304	304	300	300
r^a^	**-0.116** ^**b**^	**0.24** ^**b**^	**-0.182** ^**b**^	**-0.199** ^**b**^	-0.048	**-0.342** ^**b**^	**-0.456** ^**b**^	**-0.413** ^**b**^	**-0.448** ^**b**^	0.127
*P*	.024	< .0001	< .0001	< .0001	.343	< .0001	< .0001	< .0001	< .0001	.028
Adjusted *P*^c^	.056	< .0001	.001	.0003	.343	< .0001	< .0001	< .0001	< .0001	.056
**HIV infected, no.**	268	267	268	281	263	262	217	218	218	218
r^a^	**-0.164** ^**b**^	0.129	**-0.204** ^**b**^	**-0.35** ^**b**^	**-0.206** ^**b**^	**-0.388** ^**b**^	**-0.499** ^**b**^	**-0.515** ^**b**^	**-0.487** ^**b**^	0.114
*P*	.007	.035	.001	< .0001	.001	< .0001	< .0001	< .0001	< .0001	.092
Adjusted *P*^c^	.021	.070	.003	< .0001	.003	< .0001	< .0001	< .0001	< .0001	.092

Analyses were performed by using GeneXpert cycle threshold values and log_10_ -transformed cell counts, IL-1β concentrations, and TNF-α concentrations.

Abbreviations: IL-1β, interleukin 1β; PC, principal component; TNF-α, tumor necrosis factor α.

^a^Spearman’s ρ correlation coefficient.

^b^The adjusted *P* value is <.05.

^c^Adjusted with the Hochberg method.

PCA was used to evaluate 10 baseline CSF cytokine concentrations, to identify correlated measurements that accounted for the variance of the data set. The first component, PC1, covered 71.4% variance of the cytokine profile, PC2 covered 6.4%, PC3 covered 4.9%, PC4 covered 4.4%, and other PCs covered less (Supplementary Figure 2). In other words, the majority of the variance was reflected by just the first 2 PCs (ie, PC1 and PC2). Because all of the coefficients were located in quadrant I and quadrant IV, a positive value of PC1 implies a high concentration across all cytokines. In other words, levels of these cytokines were in strong cocorrelation with each other. Meanwhile, PC2 explains the difference in concentrations of 2 groups of cytokines: IL-13, IFN-γ, IL-10, and IL-5 versus IL-2, IL-6, and IL-1β. We used the first 2 PCs to represent the cytokine profile, to assess whether bacterial load was related to cytokine concentrations. *M. tuberculosis* loads stratified by Ct values showed a negative relationship with PC1 overall (r = −0.493 and *P* < .0001) and with respect to HIV infection status (r = −0.448 and −0.487 [*P* < .0001] for HIV-uninfected and HIV-infected groups, respectively; Table 2). *M. tuberculosis* loads were not correlated with PC2 in participants stratified by HIV status. These results suggest that participants with a high *M. tuberculosis* load tended to have high concentrations of measured cytokines. This can be seen particularly in the strong relationship between *M. tuberculosis* load and the key proinflammatory cytokines IL-1β and TNF-α (Table 2, Figure 2C and 2D, and Supplementary Figure 3). Interestingly, the CSF neutrophil count was positively correlated with PC1 regardless of HIV status (r = 0.381 and *P* < .0001 for the overall group, r = 0.385 and *P* < .0001 for the HIV-uninfected group, and r = 0.317 and *P* = .0003 for the HIV-infected group).

### Association Between *LTA4H* Genotype and *M. tuberculosis* Load

Previously, we showed that, in HIV-uninfected adults with TBM, *LTA4H* genotype was associated with CSF IL-1β, IL-2, and IL-6 concentrations, with low concentrations for genotype CC, intermediate concentrations for genotype CT, and high concentrations for genotype TT [4]. Laarhoven et al reported that the TT genotype was associated with decreased CSF culture positivity [5]. We analyzed the relationship of *LTA4H* genotype and bacterial load and found that the *M. tuberculosis* load in participants with genotype TT was slightly lower than that in those with genotype CC (*P* = .051) or CT (*P* = .015), but the linear trend test showed that the *LTA4H* genotype was not associated with the *M. tuberculosis* load overall (*P* = .302; Supplementary Figure 4).

### Relationship Between CSF *M. tuberculosis* Load, Inflammation, and New Neurological Events

Study participants were treated with intensive (n = 347) or standard (n = 345) antituberculosis treatment. As previously reported, the intensive regimen was not associated with any improvement in any measure of treatment response or outcomes, including survival [11]. During 9 months of antituberculosis treatment, new neurological events developed in 103 participants (14.9%), and the median time to new events after the start of treatment was 9 days (interquartile range, 3–43 days; Supplementary Figure 5*A*).

**Figure 3.**
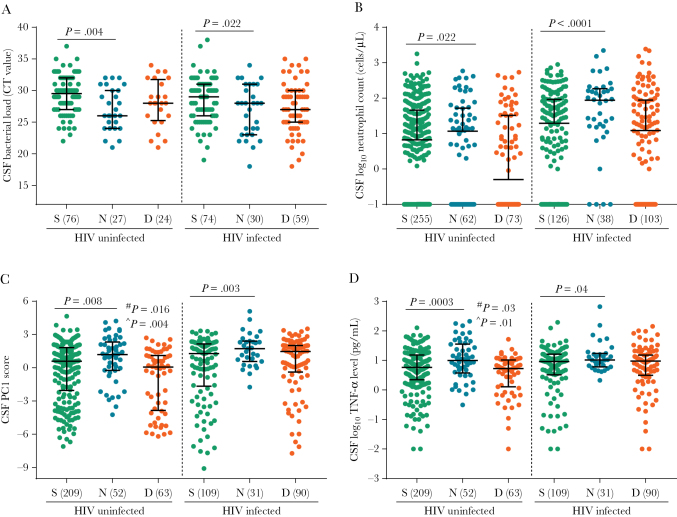
Associations between pretreatment cerebrospinal fluid (CSF) bacterial load, inflammation, and new neurological events or death. *A*, Comparison of the number of bacteria in CSF by use of the cycle threshold (Ct) value, which is inversely proportional to the bacterial load (ie, the lower the value, the higher the load) in survivors (S), those with new neurological events (N), and those who died (D) among participants with and those without human immunodeficiency virus (HIV) infection. Numbers of cases are presented in brackets. *B*, Comparison of neutrophil numbers in CSF at baseline in participants with and those without HIV infection in the S, N, and D groups. *C* and *D*, Comparison of CSF cytokine concentration principal component 1 (PC1) and tumor necrosis factor α (TNF-α) concentration in participants with and those without HIV infection in the S, N, and D groups. CSF neutrophil and cytokine data were available for 657 and 554 patients, respectively. Bars represent medians and interquartile ranges. Statistical comparisons between survival and either new neurological events or death were made using Mann-Whitney tests. ^#^, comparison of the S and D groups; ^^^, comparison of the N and D groups.

New neurological events were significantly associated with higher pretreatment *M. tuberculosis* loads in the GeneXpert-positive data set (*P* = .004 in HIV-uninfected individuals, and *P* = .022 in HIV-infected individuals; Figure 3A). In the whole data set, an elevated *M. tuberculosis* load increased the risk of new neurological events (OR, 1.68 [95% CI, 1.26–2.25; *P* = .0004] per 10-fold increase in bacterial load; Table 3). In both HIV-uninfected participants and HIV-infected participants, these events were also associated with high baseline CSF neutrophil counts, high baseline PC1 scores, and high baseline TNF-α concentrations (Figure 3B–D).

**Table 3.** Association Between Pretreatment Variables and New Neurological Events and Death, Overall and by Human Immunodeficiency Virus (HIV) Status, by Multinomial Logistic Regression Analysis

Variable	Overall (n = 692)		HIV Uninfected (n = 404)		HIV Infected (n = 288)	
	HR (95% CI)	*P*	HR (95% CI)	*P*	HR (95% CI)	*P*
**New neurological events** (*M. tuberculosis* load effect)						
*M. tuberculosis* load (per 10-fold increase)	1.68 (1.26–2.25)	.0004	2.02 (1.26–3.23)	.003	0.67 (.16–.98)	.038
*M. tuberculosis* below LOD^a^	3.12 (.96–10.1)	.057	6.83 (1.10–42.4)	.039	1.32 (.25–7.03)	.745
**New neurological events** (adjusted effect)^b^						
*M. tuberculosis* load (per 10-fold increase)^c^	1.56 (1.14–2.11)	.005	1.97 (1.18–3.28)	.009	1.43 (.95–2.15)	.082
*M. tuberculosis* below LOD^a^	2.84 (.83–9.68)	.096	7.60 (1.05–54.9)	.044	1.55 (.27–8.75)	.617
Age (per 10-y increase)	1.07 (.91–1.27)	.416	1.09 (.90–1.31)	.367	1.16 (.67–2.03)	.593
Weight (per 10-kg increase)	1.14 (.87–1.49)	.325	1.04 (.74–1.46)	.831	1.33 (.83–2.12)	.228
Glasgow coma scale (per 1-point increase)	0.77 (.71–.84)	< .0001	0.77 (.69–.85)	< .0001	0.75 (.63–.89)	.001
CSF leukocyte count (per 10-fold increase)	1.00 (.99–1.00)	.552	1.00 (.99–1.00)	.733	1.00 (.99–1.01)	.393
Intensified regimen	0.84 (.53–1.33)	.453	0.53 (.29–.96)	.037	1.76 (.80–3.90)	.159
HIV infected	1.07 (.64–1.80)	.791	…		…	
Antiretroviral therapy at enrollment	…		…		1.83 (.80–4.18)	.149
*LTA4H* genotype: CC vs TT	1.08 (.51–2.28)	.830	1.36 (.49–3.77)	.547	0.65 (.20–2.10)	.473
*LTA4H* genotype: CT vs TT	0.96 (.46–2.02)	.915	0.84 (.30–2.34)	.741	0.9 (.28–2.86)	.856
**Mortality** (*M. tuberculosis* load effect)						
*M. tuberculosis* load (per 10-fold increase)	1.49 (1.15–1.92)	.002	1.54 (.96–2.48)	.072	0.73 (.54–.99)	.045
*M. tuberculosis* below LOD^a^	3.06 (1.14–8.22)	.026	3.82 (.66–22.1)	.134	3.22 (.94–11.05)	.063
**Mortality** (adjusted effect)						
*M. tuberculosis* load (per 10-fold increase)^d^	1.25 (.94–1.68)	.127	1.43 (.82–2.50)	.207	1.14 (.79–1.64)	.480
*M. tuberculosis* below LOD^a^	2.76 (.89–8.53)	.077	2.72 (.35–21.3)	.341	2.45 (.58–10.2)	.220
Age (per 10-y increase)	1.52 (1.30–1.78)	< .0001	1.68 (1.40–2.01)	< .0001	0.91 (.59–1.41)	.675
Weight (per 10-kg increase)	0.67 (.51–.88)	.003	0.87 (.59–1.27)	.475	0.58 (.39–.88)	.010
Glasgow coma score (per point increase)	0.69 (.64–.75)	< .0001	0.71 (.64–.80)	< .0001	0.65 (.56–.75)	< .0001
CSF leukocyte count (per 10-fold increase)	1.00 (.99–1.00)	.270	0.98 (.97–1.00)	.094	1.00 (.99–1.00)	.933
Intensified regimen	0.91 (.60–1.38)	.667	0.77 (.42–1.41)	.405	1.15 (.64–2.08)	.637
HIV infected	5.50 (3.38–8.96)	< .0001	…		…	
Antiretroviral therapy at enrollment	…		…		0.80 (.43–1.50)	.487
*LTA4H* genotype: CC vs TT	2.24 (1.06–4.76)	.033	3.82 (1.02–14.3)	.046	1.66 (.64–4.35)	.297
*LTA4H* genotype: CT vs TT	1.86 (.87–3.96)	.108	2.04 (.54–7.65)	.289	1.83 (.69–4.87)	.225

Abbreviations: CI, confidence interval; CSF, cerebrospinal fluid; HIV, human immunodeficiency virus; HR, hazard ratio; LOD, limit of detection; *M. tuberculosis*, *Mycobacterium tuberculosis*.

^a^The *M. tuberculosis* load was considered below the limit of detection (LOD) if GeneXpert results were negative. The *M. tuberculosis* load covariate and indicator of LOD were used in the model.

^b^Adjusted for risk factors in the multivariate model.

^c^ *P* values were adjusted by the Hochberg method for the association of *M. tuberculosis* load and new neurological events: *P* = .045 (all patients), *P* = .075 (HIV-uninfected patients), and *P* = .823 (HIV-infected patients).

^d^ *P* values were adjusted by the Hochberg method for the association of *M. tuberculosis* load and death: *P* = .382 (all patients), *P* = .475 (HIV-uninfected patients), and *P* = .933 (HIV-infected patients).

The pathogenesis of neurological events may differ according to their timing during treatment. Therefore, we categorized participants by the median time to new neurological events (ie, <9 days or ≥9 days) and compared the bacterial load between these 2 groups. Bacterial loads were not significantly different between early and late neurological events (*P* = .196 for the HIV-uninfected group, and *P* = .061 for the HIV-infected group; Supplementary Figure 6*A*).

### Predictors of New Neurological Events

The results (Table 3) indicated that increased *M. tuberculosis* load was independently associated with new neurological events in all participants (OR, 1.56 [95% CI 1.14–2.11; *P* = .005 and adjusted *P* = .045] per 10-fold load increase). The *M. tuberculosis* load was significantly associated with new neurological events in HIV-uninfected participants (OR, 1.97; 95% CI, 1.18–3.28; *P* = .009) and with a trend in HIV-infected participants (OR, 1.43; 95% CI, .95–2.15; *P* = .082). Results stratified by HIV infection lost statistical significance after adjustment for multiple testing. GCS was also strongly associated with new neurological events (OR, 0.77 [95% CI, .71–.84; *P* < .0001] per 1-point increase). These findings highlight that pretreatment CSF *M. tuberculosis* load can be used as a predictor, together with GCS, of new neurological events occurring during TBM treatment.

### Relationship Between CSF *M. tuberculosis* Load, Inflammation, and Death

Death occurred in 192 participants (27.7%), with a median time to death of 39 days (interquartile range, 9–103 days) after the start of treatment (Supplementary Figure 5*B*). The bacterial load was not significantly associated with death, with or without neurological events, in either HIV-uninfected participants or HIV-infected participants in the GeneXpert-positive data set (Figure 3A). Although CSF neutrophil counts were similar between those who died and those who survived (Figure 3B), measured cytokine concentrations were significantly lower in those who died, with or without neurological events, among HIV-uninfected participants (Figure 3C and 3D) in the whole data set. To examine whether early death was associated with bacterial load, we categorized participants by the median time to death (ie, <39 days or ≥39 days). Bacterial loads were not significantly different between those dying early and those dying late (*P* = .731 for the HIV-uninfected group, and *P* = .099 for the HIV-infected group; Supplementary Figure 6*B*).

Taken together, our data showed that a high *M. tuberculosis* load was associated with more-severe disease at baseline and strongly predicted new neurological events after the start of treatment, but it did not influence 9-month mortality (Table 3).

## DISCUSSION

TBM is one of the most difficult forms of tuberculosis to treat, with outcomes dependent on killing the bacteria with antituberculosis drugs and controlling intracerebral inflammation. However, the relationships between bacterial numbers, inflammation, and treatment response are poorly defined, primarily because of the difficulties assessing the *M. tuberculosis* load at the site of infection. We overcame some of these difficulties by quantifying bacterial numbers in CSF by GeneXpert in a large cohort of well-characterized patients recruited into a randomized controlled trial of intensive antituberculosis therapy [11]. We found that the pretreatment CSF *M. tuberculosis* load was associated with increased CSF neutrophil numbers, enhanced inflammation, and disease severity and predicted the likelihood of new neurological events after the start of antituberculosis treatment.

The CSF bacterial load showed significant correlations with neutrophil numbers in blood and CSF specimens from both HIV-infected participants and HIV-uninfected participants. Previously, CSF neutrophil numbers have been associated with *M. tuberculosis*–positive CSF cultures [6, 18], and studies in HIV-coinfected individuals have linked the presence of *M. tuberculosis* in the CSF with a neutrophil-mediated inflammatory response and an increased risk of intracerebral IRIS [9]. Neutrophils may be protective in early *M. tuberculosis* infection [19], although animal models suggest that they may exacerbate pathology in the later stages of disease [20, 21]. Clinical studies in active tuberculosis have suggested that neutrophils may influence disease progression and outcome: higher blood neutrophil counts before treatment have been associated with increased risk of mortality in patients with tuberculosis (of whom 49.4% had pulmonary tuberculosis) [22] and slower conversion of sputum to *M. tuberculosis* negativity during therapy in pulmonary tuberculosis [23]. In transcriptional profiling of blood specimens, the signature of patients with active tuberculosis was characterized by neutrophil-driven interferon-inducible gene expression [24]. Taken together, these findings all suggest that neutrophils play an important role in tuberculosis pathogenesis.

Almost half the participants had serious clinical complications during treatment (14.9% had new neurological events, and 27% died). Surprisingly, we found that the CSF *M. tuberculosis* load influenced the likelihood of new neurological events but not mortality, suggesting different underlying mechanisms for these outcomes. In support of this assertion, current and previous data suggest that new neurological events are associated with a high concentration of CSF cytokines before the start of treatment, whereas death is associated with an attenuated inflammatory responses [4].

One of the limitations of our study was that we were unable to define the likely cause of the new neurological events, using brain imaging. However, given the nature and timing of the events and their association with increased inflammation, it is reasonable to assume that many could be defined as paradoxical treatment reactions [12]. Therefore, taken together with the recent finding by Marais et al [9], our data provide further evidence for the importance of bacterial load and neutrophils in treatment-associated inflammatory complications in adults with TBM who are or are not infected with HIV. Our findings support the hypothesis that the pathogenesis of IRIS and paradoxical reactions are similar, resulting from excessive neutrophil-mediated inflammation and driven by *M. tuberculosis* load. Drugs with the potential to target neutrophils (eg, roflumilast, ibuprofen, and doxycycline [12]) may be more effective than corticosteroids in the treatment of these common complications.

An additional limitation of our study was that we could not follow the decline of bacterial loads after the start of treatment. Even before the start of antituberculosis treatment, CSF bacillary loads are very low. In our participants, pretreatment bacillary loads in CSF specimens were at least 100-fold lower than loads reported in sputum specimens from patients with pulmonary tuberculosis [25]. Furthermore, repeated CSF sampling early in treatment is rarely clinically justifiable, which further restricts the information available. We routinely sample CSF after 30 and 60 days of therapy, by which time bacterial loads are almost always below the detection threshold.

In summary, our data shed new light on the pathogenesis of TBM and suggest divergent mechanisms that lead to death and neurological events occurring after the start of treatment. Death from TBM is associated with an attenuated inflammatory response at the start of treatment, with reduced CSF leukocytes and cytokine concentrations, compared with those in survivors. In contrast, patients with high pretreatment CSF bacterial loads, cytokines, and neutrophils are more likely to experience new neurological events or paradoxical inflammatory complications. These findings may have important implications for the selection of future host-directed therapies.

## Supplementary Material

Supplementary Material

## Notes

***Acknowledgments.*** We thank the participants in the study and the physicians and nurses at HTD and PNT hospitals who were responsible for their care and follow-up.

***Financial support.*** This work was supported by the Wellcome Trust (206724/Z/17/Z to N.T.T.T and 106680/B/14/Z to G.T).

***Potential conflicts of interest.*** All authors: No reported conflicts.
