# Supplementary material for: Pretreatment Cerebrospinal Fluid Bacterial Load Correlates With Inflammatory Response and Predicts Neurological Events During Tuberculous Meningitis Treatment
Source: J Infect Dis. 2018 Oct 9;219(6):986–95. doi: 10.1093/infdis/jiy588 (PMC6386814; doi:10.1093/infdis/jiy588)
Supplement: Supplementary Material [file jiy588_suppl_jiy588_suppl_supplementary_material.docx]

**Table S1. Baseline clinical characteristics of all 692 TBM participants by HIV infection**

| **Characteristic** | **All** | | **HIV-uninfected** | | **HIV-infected** | | **p values** |
| --- | --- | --- | --- | --- | --- | --- | --- |
|  | **n** | **Summary statistic** | **n** | **Summary statistic** | **n** | **Summary statistic** |  |
| Age (years) – median (IQR) | 692 | 35 (29,46) | 404 | 41 (28,55) | 288 | 33 (30,37) | < 0.0001 |
| Male sex – no. (%) | 692 | 475 (68.6) | 404 | 239 (59.2) | 288 | 236 (81.9) | <0.0001 |
| Weight (kg) – median (IQR) | 692 | 48 (44,54.5) | 404 | 49 (45,55) | 288 | 47 (42.75,52.05) | 0.009 |
| Duration of illness (days) – median (IQR) | 692 | 15 (10,30) | 404 | 15 (10,23) | 288 | 15 (8,30) | 0.997 |
| Glasgow coma score | 692 | 15 (12,15) | 404 | 15 (13,15) | 288 | 15 (12,15) | 0.612 |
| Treatment arm- *Standard arm* – no. (%) | 692 | 345 (49.9) | 404 | 202 (50.0) | 288 | 143 (49.7) | 0.928 |
| ^#^BMRC Grade – no. (%) | 692 |  | 404 |  | 288 |  | 0.168 |
| - Grade 1 |  | 266 (38.4) |  | 145 (35.9) |  | 121 (42.0) |  |
| - Grade 2 |  | 302 (43.6) |  | 188 (46.5) |  | 114 (39.6) |  |
| - Grade 3 |  | 124 (17.9) |  | 71 (17.6) |  | 53 (18.4) |  |
| Diagnostic tests |  |  |  |  |  |  |  |
| ­CSF-Smear - *Positive –* no. (%) | 684 | 257 (37.6) | 399 | 121 (30.3) | 285 | 136 (47.7) | < 0.0001 |
| ­CSF-MGIT culture - *Positive –* no. (%) | 682 | 281 (41.2) | 400 | 125 (31.3) | 282 | 156 (55.3) | < 0.0001 |
| ­CSF-GeneXpert- *Positive –* no. (%) | 692 | 293 (42.3) | 404 | 128 (31.7) | 288 | 165 (57.3) | < 0.0001 |
| *Diagnostic category – no. (%) | 692 |  | 404 |  | 288 |  | < 0.0001 |
| - Definite TBM |  | 393 (56.8) |  | 188 (46.5) |  | 205 (71.2) |  |
| - Probable TBM |  | 172 (24.9) |  | 117 (29.0) |  | 55 (19.1) |  |
| - Possible TBM |  | 127 (18.4) |  | 99 (24.5) |  | 28 (9.7) |  |
| Blood - median (IQR) |  |  |  |  |  |  |  |
| -Sodium (mmol/l) | 634 | 128 (123,133) | 370 | 129 (125,133) | 264 | 126 (122,131) | < 0.0001 |
| -Leucocytes total (x 10^6^ cells/ml) | 653 | 9.1 (6.8,12.0) | 383 | 10.3 (8.2,13.1) | 270 | 7.17 (5.4,10.0) | < 0.0001 |
| -Neutrophils (%) | 653 | 80 (72,87) | 383 | 81 (73,87) | 270 | 78 (71,85) | 0.02 |
| -Lymphocytes (%) | 653 | 11 (7,17) | 383 | 11 (7,17) | 270 | 12 (7,17) | 0.381 |
| -CD4 cell count (x 10^3^ cells/ml) | - | - | - | - | 249 | 40 (15,108) | - |
| Cerebrospinal fluid - median (IQR) |  |  |  |  |  |  |  |
| -Leucocytes total (x 10^3^ cells/ml) | 686 | 116 (35,284) | 402 | 114 (40,264) | 284 | 122 (27,351) | 0.493 |
| -Neutrophils (%) | 655 | 10 (0,32) | 390 | 5 (0,20) | 265 | 15 (0,45) | < 0.0001 |
| -Lymphocytes (%) | 656 | 90 (68,100) | 390 | 95 (80,100) | 266 | 82.5 (51,100) | < 0.0001 |
| -Protein (g/l) | 666 | 1.2 (0.6,1.9) | 391 | 1.1 (0.6,1.7) | 275 | 1.4 (0.8,2.2) | < 0.0001 |
| -Glucose (mmol/l) | 666 | 1.9 (1.3,2.7) | 391 | 2.0 (1.3,2.9) | 275 | 1.8 (1.1,2.5) | 0.002 |
| -Lactate (mmol/l) | 638 | 4.8 (3.5,6.5) | 374 | 4.8 (3.6,6.4) | 264 | 4.89 (3.6,6.6) | 0.99 |

All summary statistics are absolute counts (%) for categorical variables and median (inter-quartile range = IQR) for continuous data. n refers to the number of patients with non-missing data for the corresponding variable.

p values are descriptive only and based on χ^2^ test for categorical data and Mann-Whitney test for continuous data.

^#^BMRC denotes modified British Medical Research Council criteria. Grade I indicates a GCS of 15 with no neurologic signs (baseline), grade II a score of 11 to 14 (or 15 with focal neurologic signs), and grade III a score of 10 or less.

*Diagnostic categories were assigned according to the consensus case definition.^1^ Patients with an unlikely diagnosis of tuberculous meningitis had a score of <6. Confirmed other diagnosis was only made based on microbiological evidence (Marais et al.; The Lancet Infectious diseases. 2010; 10(11): 803-12)


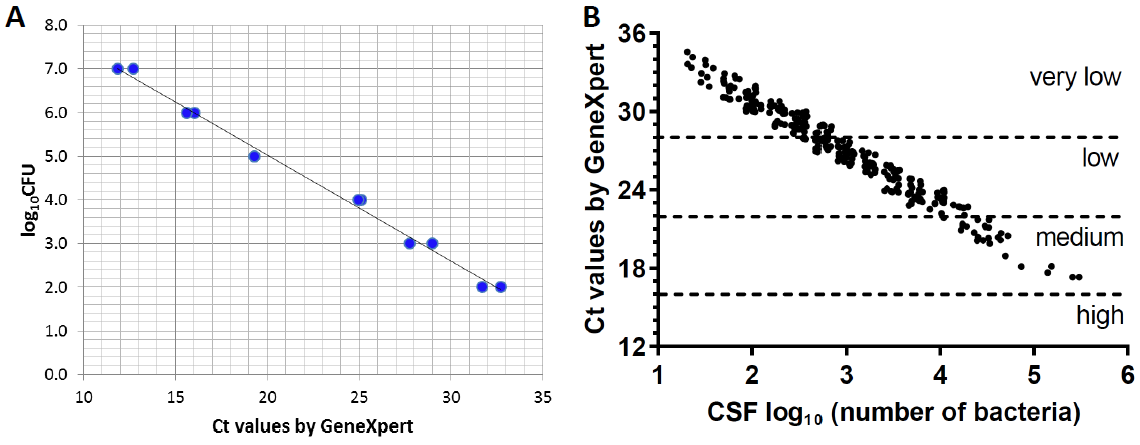


**Figure S1. Conversion of Ct values by GeneXpert to bacterial numbers**

(A) Standard curve of Ct values by GeneXpert versus log_10_ of colony forming units (CFU). BCG strain was cultured, quantified by Miles and Misra method (The Journal of Hygiene. 1938;38(6):732-49) and tested with the GeneXpert MTB/RIF assay. The standard curve obtained from DNA extracted from 10-fold dilutions of BCG from 10^1^ to 10^7^ CFU showed coefficient of determination r^2^=0.992 and the number of bacteria was converted from the Ct value by using the equation: y = - 0.243 x + 9.880. (B) Ct values of CSF samples from TBM patients in the study were converted to number of bacteria using the standard curve in Figure 1A. Bacterial numbers of very low bacterial loads range from 8 to 1.2×10^3^ (median = 0.2×10^3^/ reaction), low loads range from 1.2×10^3^ to 34.7×10^3^ (median = 3.7×10^3^/ reaction) and medium loads range from 34.7×10^3^ to 324.6×10^3^ (median = 34.7×10^3^/ reaction). Volumes of CSF per reaction range from 1.0-1.7 ml (one third of 3-5 ml sample) depending on the sample collected from each patient.


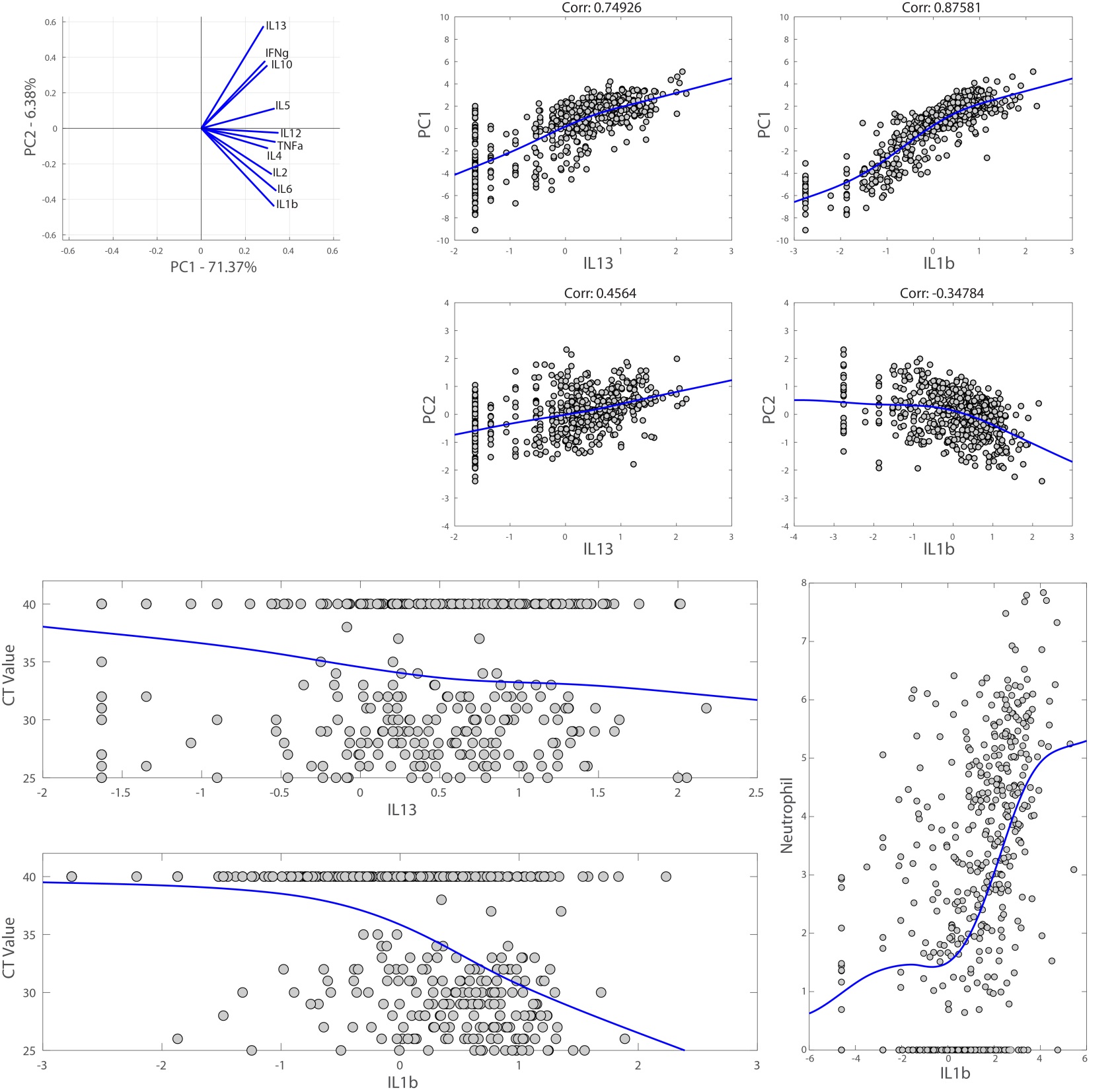


**Figure S2.** **Coefficients of ten cytokines by principal component analysis** **(PCA)**

Baseline concentrations of the ten CSF cytokines, consisting of IL-1β, IL-2, IL-4, IL-5, IL-6, IL-10, IL-12p70, IL-13, IFN-γ, TNF-α, were analysed using PCA. The center of the panel corresponds to the data point that has average cytokine value, the PC1 shows the scale of the cytokine concentration representing 71.37% of the variance, and PC2 contributing 6.38% of the variance. Because all of the coefficients are located in quadrants I and IV, a positive value of PC1 implies high concentration across all cytokines. In other words, these cytokines were in strong co-correlation with each other. Meanwhile the PC2 explains the difference in cytokine concentrations of two groups, which are IL-13, IFN-γ, IL-10 and IL-5 in group 1, and other cytokines in group 2.


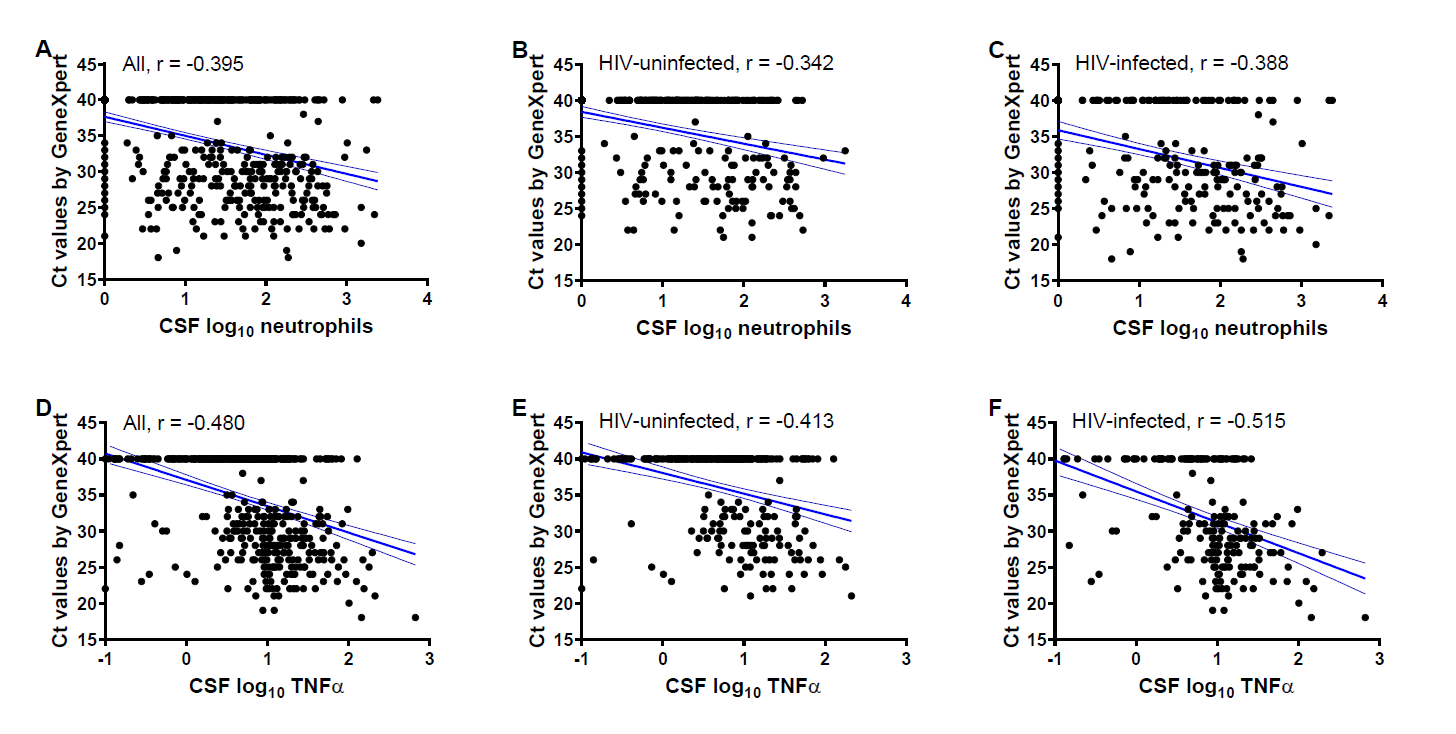


**Figure S3. Relationship of *Mtb* load and neutrophils and TNFα in CSF from TBM patients by Spearman correlation**

Correlations between *Mtb* load and CSF neutrophils in all TBM patients (A), HIV-uninfected (B) and HIV-uninfected (C). Correlations between *Mtb* load and CSF TNFα concentration in all TBM patients (D), HIV-uninfected (E) and HIV-uninfected (F).

Analyses were performed using GeneXpert Ct values and log_10_ -transformed cell counts, and TNF-α concentration.

r: Spearman’s rho correlation coefficient, p values adjusted by the Hochberg method < 0.0001 for all these tests. Best-fit

regression lines are shown with 95% confidence intervals.


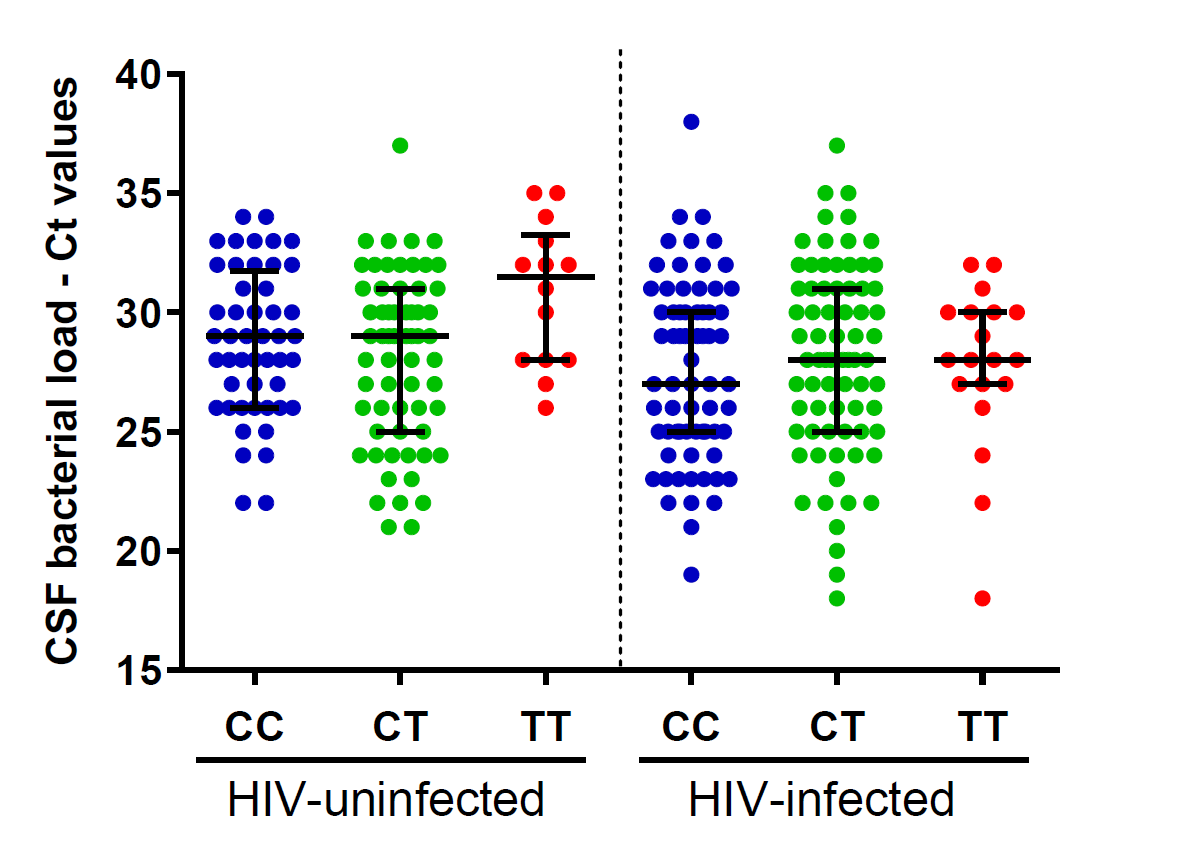


**Figure S4. *LTA4H* genotype and pre-treatment *Mtb* load from TBM adults stratified by HIV infection**

Data are for *LTA4H* genotype with 48 CC, 62 CT and 73 TT in HIV-uninfected and 68 CC, 73 CT and 19 TT in HIV-infected. Statistical analyses by Mann-Whitney between two groups, TT vs CC, p=0.051 and TT vs CT, p=0.015 in HIV-uninfected; linear trend test for 3 genotypes, p = 0.302.


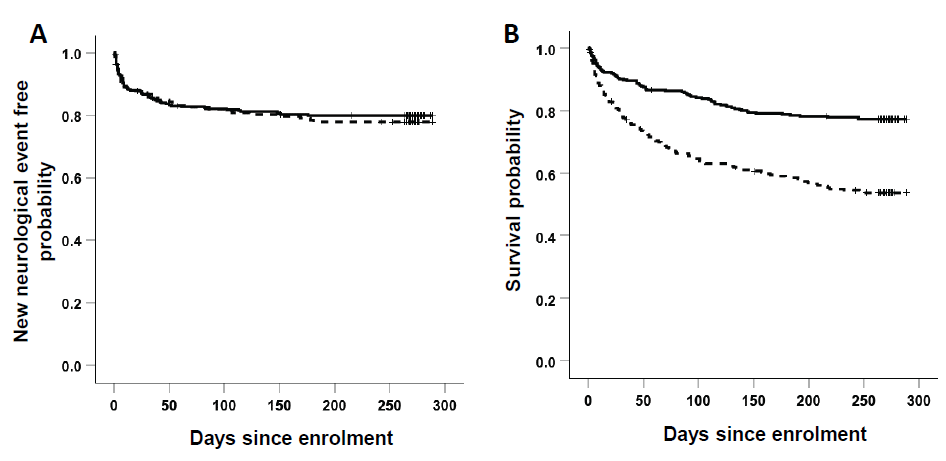


**Figure S5. Kaplan-Meier curves of new neurological events and survival stratified by HIV status**

(A) New neurological events. (B) Survival. Solid lines represent HIV-uninfected patients; dashed lines represent HIV-infected patients. Data are for 397 survival, 103 new neurological events and 192 death.


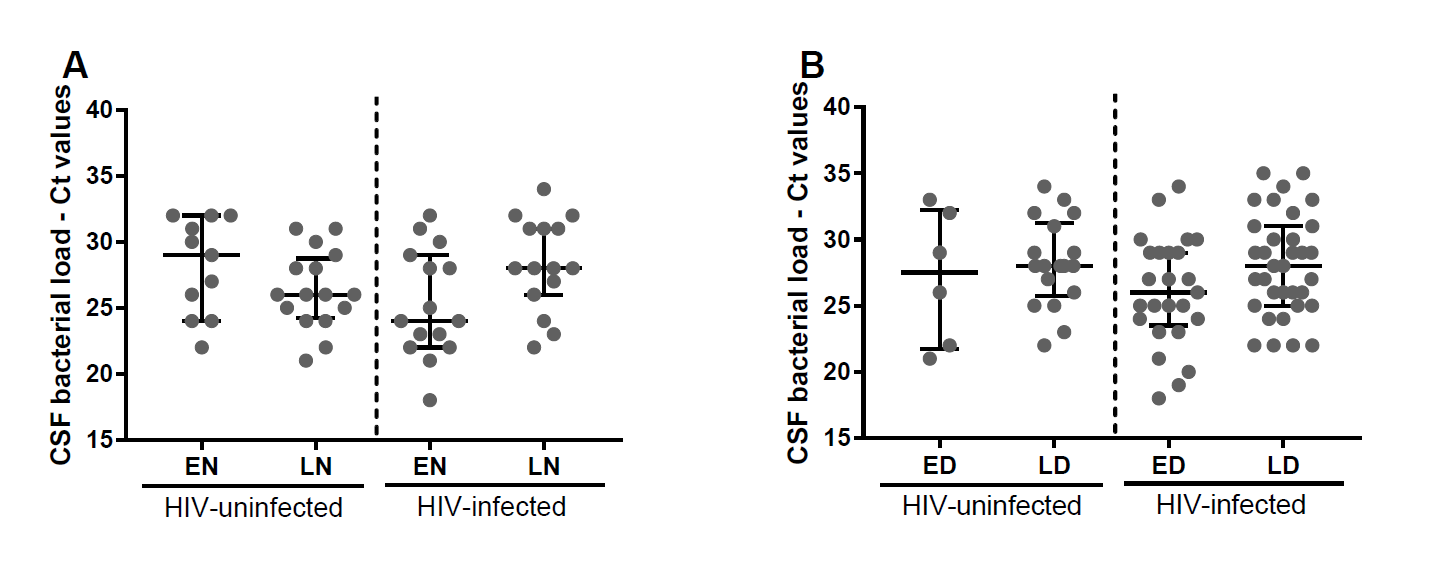


**Figure S6.** **Associations between pre-treatment CSF bacterial load and time to new neurological events or death**

(A) Bacterial load in early new neurological events (EN, < 30 days) and late neurological events (LN, ≥ 30 days) in 27 HIV-uninfected patients (12 EN, 15 LN) and in 30 HIV-infected patients (18 EN, 12 LN). (B) Bacterial load in early dead (ED, < 90 days) and late dead (LD, ≥ 90 days) in 24 HIV-uninfected patients (11 ED, 13 LD) and in 59 HIV-infected patients (40 ED, 19 LD). Bars in plots represent median and inter-quantile range values. Statistical comparisons between early versus late new neurological events or early versus late death were made using Mann-Whitney tests, all p-values > 0.05.
